# Electrical tunability of terahertz nonlinearity in graphene

**DOI:** 10.1126/sciadv.abf9809

**Published:** 2021-04-07

**Authors:** Sergey Kovalev, Hassan A. Hafez, Klaas-Jan Tielrooij, Jan-Christoph Deinert, Igor Ilyakov, Nilesh Awari, David Alcaraz, Karuppasamy Soundarapandian, David Saleta, Semyon Germanskiy, Min Chen, Mohammed Bawatna, Bertram Green, Frank H. L. Koppens, Martin Mittendorff, Mischa Bonn, Michael Gensch, Dmitry Turchinovich

**Affiliations:** 1 Helmholtz-Zentrum Dresden-Rossendorf, Bautzner Landstraße 400, 01328 Dresden, Germany.; 2 Fakultät für Physik, Universität Bielefeld, Universitätsstr. 25, 33615 Bielefeld, Germany.; 3 Catalan Institute of Nanoscience and Nanotechnology (ICN2), BIST and CSIC, Campus UAB, 08193, Bellaterra (Barcelona), Spain.; 4 Institut de Ciencies Fotoniques (ICFO), The Barcelona Institute of Science and Technology, Barcelona, Spain.; 5 Institució Catalana de Recerça i Estudis Avancats (ICREA), 08010 Barcelona, Spain.; 6 Fakultät für Physik, Universität Duisburg-Essen, Lotharstraße 1, 47057 Duisburg, Germany.; 7 Max-Planck-Institut für Polymerforschung, Ackermannweg 10, 55128 Mainz, Germany.; 8 Institut für Optische Sensorsysteme, DLR, Rutherfordstraße 2, 12489 Berlin, Germany; 9 Institut für Optik und Atomare Physik, Technische Universität Berlin, Strasse des 17. Juni 135, 10623 Berlin, Germany.

## Abstract

Graphene is conceivably the most nonlinear optoelectronic material we know. Its nonlinear optical coefficients in the terahertz frequency range surpass those of other materials by many orders of magnitude. Here, we show that the terahertz nonlinearity of graphene, both for ultrashort single-cycle and quasi-monochromatic multicycle input terahertz signals, can be efficiently controlled using electrical gating, with gating voltages as low as a few volts. For example, optimal electrical gating enhances the power conversion efficiency in terahertz third-harmonic generation in graphene by about two orders of magnitude. Our experimental results are in quantitative agreement with a physical model of the graphene nonlinearity, describing the time-dependent thermodynamic balance maintained within the electronic population of graphene during interaction with ultrafast electric fields. Our results can serve as a basis for straightforward and accurate design of devices and applications for efficient electronic signal processing in graphene at ultrahigh frequencies.

## Teaser

We turn graphene from an inert electronic material to the most nonlinear material for the terahertz range using a small voltage.

## INTRODUCTION

The development of efficient broadband electronic frequency multipliers and modulators operating at the technologically important terahertz frequency range and under normal ambient conditions is of great relevance, but also challenging (*1*–*5*). These technologies require the integration of a highly nonlinear material in an electronic device with the possibility to control its nonlinear behavior. In addition, compatibility between the device constituents is crucial. These requirements are not easy to meet, especially in simple electronic circuits (*4*–*6*). Here, we demonstrate the possibility of accomplishing these requirements in a graphene-based device. A straightforward and efficient wide-range control of the terahertz nonlinearity of graphene is enabled by electrical gating of only a few volts.

One of the essential technological consequences of the Dirac-type electronic bandstructure of graphene (*7*, *8*) is its extremely nonlinear response to electric fields in the terahertz frequency range, which persists at room temperature and under normal ambient conditions. Graphene is a highly efficient terahertz nonlinear absorber, capable of displaying a nonlinear power transmission modulation of about 50% per single monolayer (*9*–*12*). It is also an extremely efficient terahertz frequency multiplier, allowing for the straightforward generation of multiple terahertz harmonics (*12*–*15*). Such a nonlinear response is directly attainable with quite moderate input electric fields, oscillating at frequencies not exceeding a few hundred gigahertz and with a field strength in the range of 10 to 100 kV/cm, which is even lower than typical channel fields in the current generation of high-speed transistors (*4*, *16*. Conveniently, graphene is also compatible with complementary metal-oxide semiconductor (CMOS) technology (*6*, *17*). This suggests the possibility to develop hybrid graphene-CMOS technology, with the subterahertz CMOS device acting as a reliable and cost-effective pump source, providing the input signal for the efficient nonlinear processing in graphene at terahertz frequencies. One of the straightforward applications of such a technology is all-electronic terahertz frequency mixing or upconversion of the subterahertz CMOS–generated signals into the terahertz range, with the potential to outcompete the current generation of diode-based ultrahigh frequency mixers (*1*–*3*, *18*, *19*) in terms of conversion efficiency, operation bandwidth, compactness, and cost.

Recent experiments on terahertz high-harmonic generation (HHG) in graphene directly provided the measurement of its nonlinear coefficients up to the seventh order (*14*). These nonlinear coefficients were found to exceed the respective coefficients of all other known electronic materials by many orders of magnitude (*12*, *14*). In a nonoptimized terahertz HHG experiment in (*14*), the field conversion efficiency from the driving subterahertz electric signal to terahertz higher harmonics of η ~ 1% was demonstrated in just a single–atomic layer graphene sample. Such a strong terahertz nonlinearity of graphene is attributed to the collective thermodynamic response of its background free-carrier population (electrons or holes) to the driving terahertz field (*11*, *12*, *14*). Increasing the density of these free carriers in graphene (or equivalently, its Fermi energy *E*_F_) enhances the power absorption of the terahertz driving field and consequently provides a control knob that allows for tuning the nonlinear response. On the other hand, too high carrier density and hence the strongly metallic phase of graphene with increased electronic heat capacity will diminish the material’s thermodynamic nonlinearity. This suggests the existence of optimal doping in graphene, favoring its terahertz nonlinearity, which can be controlled externally. Here, we demonstrate such an efficient control of graphene nonlinearity by varying its free carrier density by electrical gating.

Being only one atomic layer thick and having a gapless bandstructure, graphene is a material for which wide tuning of the Fermi energy is easily achievable using a small gating voltage of only a few volts, not only in one band but also between the valence and conduction bands across the Dirac point. This offers a simple route to control the terahertz nonlinearity of graphene. Here, we demonstrate the effects of the carrier density (or the material Fermi energy, *E*_F_) on terahertz saturable absorption (SA) and HHG, as well as the associated nonlinear carrier dynamics in gated graphene. We achieve this using nonlinear terahertz spectroscopy using single- and multicycle terahertz driving fields with a peak electric field strength reaching 80 kV/cm. Within the experimentally tested range of *E*_F_, increasing from ~50 to 200 meV relative to the Dirac point, transient changes in the terahertz field transmission of graphene in response to a terahertz driving field of 80 kV/cm are enhanced by a factor of ~70. For the same range of increasing *E*_F_, an enhancement in the power conversion efficiency in third terahertz harmonic generation by almost two orders of magnitude has been achieved.

### Physical picture of gate-tunable terahertz nonlinearity

**Fig. 1:**
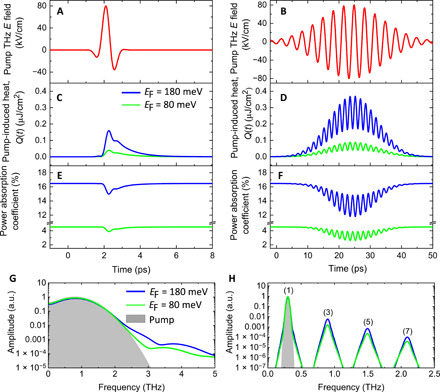
Thermodynamic model calculations for the Fermi energy dependence of the terahertz nonlinearity of graphene. (**A** and **B**) Driving single-cycle and multicycle terahertz fields, respectively. (**C** and **D**) and (**E** and **F**) The associated terahertz field–induced heat and SA, respectively, for two-doping levels corresponding to Fermi energies of 80 and 180 meV. The terahertz-induced heat (or equivalently the rise in the electron temperature) leads to a reduction and a temporal modulation of both the intraband conductivity, σ(*t*) and the power absorption coefficient α(*t*), as shown in (C) and (D). This results in a nonlinear terahertz field–induced current *J*(*t*) = σ(*t*, *E*_THz_)*E*_THz_(*t*) in the graphene layer, yielding terahertz field–induced transparency (SA) and electromagnetic reemission at higher odd-order harmonics. Both the terahertz-induced heat and SA become more pronounced when the doping concentration (the Fermi energy, *E*_F_) increases, scaling nearly with $E_{F}^{2}$. (**G** and **H**) The spectral amplitudes of the terahertz fields transmitted through graphene relative to the pump field (gray background), for the tested Fermi energies of 80 and 180 meV. a.u., arbitrary units.

The nonlinear interaction between free carriers in graphene and the field of a driving terahertz signal resembles a thermodynamic process exhibiting an interplay of heating-cooling dynamics (*11*, *12*, *14*), as illustrated in Fig. 1 for two different terahertz pulse shapes: single-cycle (Fig. 1A) and a multicycle (Fig. 1B) pulses. The terahertz energy absorbed in graphene by the free carriers via the intraband optical conductivity mechanism is quasi-instantaneously (on a sub–50-fs time scale) converted into electronic heat, corresponding to a rise in the carrier temperature and consequently to a suppression of both the THz conductivity and power absorbance (i.e., SA) of graphene (see Fig. 1, C to F). This heating process is then followed by a slower, picosecond-timescale carrier cooling process, occurring via emission of optical and acoustic phonons, which then restores the initial high conductivity of graphene. The interplay of the carrier heating and cooling processes during the interaction of graphene with the driving terahertz signal results in a temporal modulation of both the terahertz conductivity and the instantaneous terahertz absorbance (see Fig. 1, C to F). This leads to a nonlinear temporal deformation of the driving terahertz field passing through graphene, which eventually results in a spectral broadening for the single-cycle pulse, as shown in Fig. 1G, and a highly efficient terahertz frequency multiplication, i.e., terahertz higher odd-order harmonic generation for the multicycle pulse, as shown in Fig. 1H (see also the Supplementary Materials for further information).

Because the electronic terahertz nonlinearity of graphene is facilitated by the absorption of the driving terahertz field by the free carriers and the subsequent thermodynamic process involving the electronic and phononic subsystems, it is the density of these free carriers that provides the key to controlling the terahertz nonlinearity in this material. That is, the terahertz nonlinearity relies on the presence of free carriers, and increasing the free carrier density increases the power absorption and leads to a stronger nonlinear response (scaling approximately with the square of the Fermi energy, *E*_F_), as shown in Fig. 1 (C to H). However, in the regime where the density becomes extremely high (likely above *E*_F_ = 200 meV), the rise in the carrier temperature becomes modest, as the absorbed terahertz energy is to be shared among a very large number of carriers, leading to a weaker nonlinear response. Hence, there is an optimal carrier density that provides the largest nonlinearity (see the Supplementary Materials for further information).

To examine the degree of control over the nonlinearity that we can expect, we first consider two levels of carrier densities corresponding to *E*_F_ of 80 and 180 meV and simulate two nonlinear experiments. We use two different terahertz waveforms, single-cycle broadband and multicycle quasi-monochromatic terahertz pulses, as shown in Fig. 1 (A and B), respectively, similar to the terahertz signals used in our experiments, with identical peak field strengths of 80 kV/cm. We see from Fig. 1 (C to H) that for both cases of terahertz driving signals, the nonlinear terahertz field–induced effects in graphene are more pronounced for the larger *E*_F_.

Another important feature shown in Fig. 1 is that for the case of multicycle quasi-monochromatic driving terahertz signal, the modulation effect in graphene is generally stronger both in amplitude and in temporal dynamics, as compared to the excitation with the single-cycle pulse, at the same value of *E*_F_ and peak electric field strength in the signal. This is clearly visualized in Fig. 1 (G and H) through the spectra of the terahertz signals transmitted through the graphene layer. The reason for the stronger nonlinear modulation of terahertz properties of graphene in case of multicycle driving field is that (i) the power carried by the multicycle pulse is larger, assuming the same peak field as for the single-cycle and (ii) the accumulated electronic heat persists more than a few hundred femtoseconds, which, in turn, allows for enhancing the electronic interaction with the longer-lasting multicycle driving signal. This presents novel opportunities for the efficient nonlinear signal processing of quasi-monochromatic terahertz electronic signals.

Our calculations based on the thermodynamic model presented here use a single-band picture, in which only intraband free carrier dynamics, subjected to the conservation of carrier density within the terahertz-excited band, is considered (i.e., either electrons in the conduction band or holes in the valence band). Whereas this approach is strictly valid for graphene with a large *E*_F_, it is expected to be less accurate for the situation with *E*_F_ < *k*_B_*T*_e_, where a two-band thermalization picture should instead be considered. Here *k*_B_ is the Boltzmann constant and *T*_e_ is the carrier temperature. However, we use the single-band approach universally for the whole range of tested Fermi energies, because it very well reproduces the entirety of our experimental observations using a minimum of adjustable parameters (namely, only the proportionality constant between the electron energy and electron scattering time). At the same time, the two-band model would require the introduction of additional adjustable parameters (see the Supplementary Materials).

### Sample and nonlinear terahertz spectroscopy

**Fig. 2:**
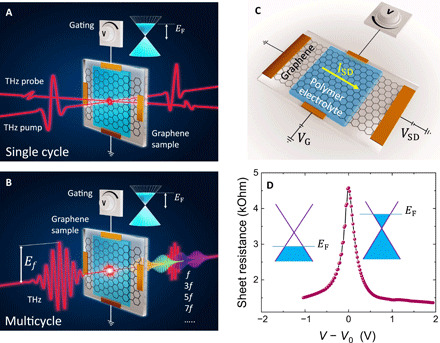
Schematics of the terahertz experiments and the gated graphene sample. (**A**) TPTP experiment with gated graphene. The electric field of the terahertz pump is vertically polarized and has a peak field strength up to 80 kV/cm, while the electric field of the terahertz probe is horizontally polarized with a peak field strength of less than 1 kV/cm. (**B**) Nonlinear terahertz-TDS, using multicycle quasi-monochromatic terahertz pulses oscillating at a fundamental central frequency of 0.3 THz and having a peak electric field up to 80 kV/cm. The transmitted field through the graphene sample consists of higher odd-order harmonics in addition to the fundamental frequency. Note that *E*_F_ refers to the Fermi energy of the graphene sample, while *E_f_* refers to the peak electric field of the driving THz signal oscillating at the fundamental frequency *f*. (**C**) The gated graphene sample device in which the graphene film acts as a channel between source and drain electrodes subjected to a constant potential difference of 0.2 mV. The graphene film is covered on top by an electrolyte subjected to a varying gating voltage to tune the Fermi level of the graphene layer. (**D**) Experimentally determined gating response of the graphene sample given by the sheet resistance of the graphene film as a function of the gating voltage relative to the voltage *V*_0_ corresponding to the minimum Fermi energy in the vicinity of the Dirac point, exhibiting a maximum resistance. Positive *V* − *V*_0_ leaves electrons in the graphene layer with the Fermi level elevated in the conduction band, while hole doping with the Fermi level pinning the valence band is induced by negative *V* − *V*_0_.

On the basis of our simulations that predict a large gate tunability of the nonlinear response in both single-cycle and multicycle cases, we have conducted two types of nonlinear terahertz spectroscopy experiments on our electrically gated graphene sample: (i) table top laser–based intense terahertz pump–terahertz probe (TPTP) spectroscopy using single-cycle broadband terahertz pulses, as shown in Fig. 2A, and (ii) accelerator-based nonlinear terahertz time-domain spectroscopy (nonlinear terahertz-TDS) with multicycle quasi-monochromatic terahertz fields, as shown in Fig. 2B. The former provided the measurements of the terahertz field–induced SA and the associated carrier (temperature) dynamics, while the latter enabled the observations of terahertz HHG. The graphene sample is composed of a chemical vapor deposition (CVD)–grown single-layer graphene on a 1-mm-thick infrasil quartz substrate and is connected at two opposite edges to two electrodes acting as source and drain. The gating is enabled by another pair of electrodes and a polymer electrolyte [LiClO_4_:polyethylene oxide (PEO)] deposited on the graphene layer and the gate electrodes, as shown in Fig. 2C. The Fermi energy of graphene is then varied by a small gate voltage in the range between −2 to +2 V (see Materials and Methods for further information about the experiments and the sample preparation).

Figure 2D shows the gating response of the graphene sample, indicated by the graphene sheet electrical resistance, retrieved by measuring the source-drain current through the graphene layer at a drain voltage of 10 mV, as a function of the gating voltage. The initial doping of the graphene layer (doping at zero gate voltage) was p-type, as usually provided unintentionally by chemical residues during the preparation process and due to interaction with the substrate (*12*, *20*, *21*), and the deposited polymer electrolyte. Here, a nonzero gate voltage *V*_0_ has compensated for this hole doping and shifted the Fermi energy to a minimum near the Dirac point, which corresponds to the peak resistance of the sample in Fig. 2D. We note here that *E*_F_ is considered relative to the Dirac point and is never uniformly zero at ∣*V* − *V*_0_∣ = 0 due to inhomogeneity in the doping and gating field across the graphene layer, as well as a finite thermal broadening of the electron distribution, given *k*_B_*T* = 26 meV at room temperature *T* = 300 K, resulting in the finite peak resistance at ∣*V* − *V*_0_∣ = 0. Increasing the absolute gating voltage away from *V*_0_ leads to an increase in the absolute value of *E*_F_, whether for electrons or holes, as shown in Fig. 2D. Quantitatively, *E*_F_ is related to the free carrier density, *N*_c_, through $E_{F}=\hbar v_{F}\sqrt{\pi N_{c}}$, where ℏ is the reduced Planck’s constant and *v*_F_ = 1 × 10^6^ m/s is the Fermi velocity in graphene.

## RESULTS AND DISCUSSION

### Saturable absorption

**Fig. 3:**
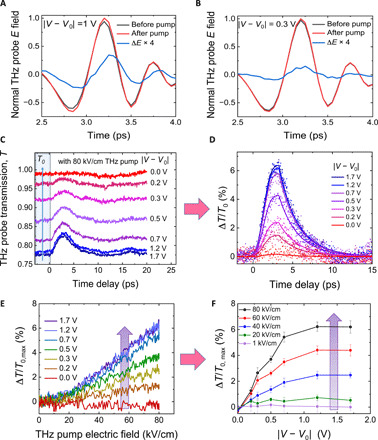
Dependence of terahertz field–induced SA on the gating voltage revealed by TPTP spectroscopy. (**A** and **B**) The terahertz probe fields before (black) and after (red) intense terahertz pump excitation, normalized to the peak field after excitation with pump peak electric field (80 kV/cm), and their difference (blue) multiplied by four for clarity, for gating voltages of 1 and 0.3 V, respectively. (**C**) The as-measured peak field transmission of the probe pulses at various gating voltages when the graphene is pumped at 80 kV/cm. *T*_0_ is the background probe transmission of the graphene sample before the pump, which increases by decreasing the doping (gating) level. (**D**) The terahertz probe differential transmission Δ*T*/*T*_0_ = (*T* − *T*_0_)/*T*_0_, where *T* is the probe transmission after the pump, as a function of the pump-probe delay time, obtained by analysis of the experimental data of (C). The dots represent the experimental results, while the solid lines are guides for the eye. (**E**) The peak of the probe differential signal Δ*T*/*T*_0_ as a function of the terahertz pump field at various doping levels and (**F**) the peak of Δ*T*/*T*_0_, extracted from (E), as a function of the gating voltage at some selected pump peak electric fields. The pink arrows indicate that the data in (D) and (F) are obtained by analysis of data from (C) and (E), respectively, while the violet arrows in (E) and (F) indicate increase in Δ*T*/*T*_0_ with the gate voltage and the driving field, respectively.

Figure 3 shows both the as-measured and processed results of the single-cycle TPTP experiment, demonstrating the efficient control of the nonlinear terahertz transmission of graphene using a small gating voltage of 2 V at maximum. Here, we refer to ∣*V* − *V*_0_∣ simply as the gating voltage. The graphene sample was excited by terahertz pump pulses with peak electric field strength ranging from 1 to 80 kV/cm. The effects induced by the terahertz pump were probed by a weak terahertz probe pulse, at various values of *E*_F_ controlled by the gating voltage. Figure 3 (A and B) shows the terahertz probe pulses transmitted through the graphene sample with and without the intense terahertz pump at 80 kV/cm for two different gating voltages. After the pump, the probe transmission increases, indicating terahertz absorption bleaching (or equivalently SA) in graphene, which corresponds to a reduction of the graphene terahertz conductivity. This effect becomes more pronounced with increasing the gating voltage (the increase in transmission of the “red” pulse relative to the “black” pulse is more pronounced in Fig. 3A compared to the situation in Fig. 3B). This is further clarified by the difference pulses shown in blue (∆*E* = *E*_red_ − *E*_black_, multiplied by four for clarity) that show a larger pump-induced change in transmission for the case of the larger gating voltage.

Figure 3C shows the as-measured transmission of the probe peak field as a function of the pump-probe delay time for various gating voltages. We note that the background probe transmission *T*_0_ before the pump decreases with the gating voltage due to increasing absorption when the carrier density increases by gating. With the intense terahertz pump, a transient increase in the transmission occurs. This nonlinearity becomes much more pronounced at a larger gating voltage. The secondary smaller features observed at ~16.5 ps in Fig. 3C are due to internal round-trip etalon reflection within the quartz substrate. Figure 3D shows the analyzed terahertz differential transmission Δ*T*/*T*_0_ of the terahertz probe obtained from the data of Fig. 3C, revealing that the nonlinear terahertz field transmission of graphene is modulated from a minimum of less than 0.1% to ~7%, i.e., by 70 times, when a gating voltage of only 2 V is applied. This demonstrates an efficient, straightforward control of the graphene nonlinearity with a very modest gating voltage.

We further observe from Fig. 3D that the transient increase in transmission reaches a peak after ~2 ps and is then followed by an exponential decay over a few picoseconds. The dots represent the experimental data from Fig. 3C, while the solid lines guide the eye (see the Supplementary Materials for further information). This temporal evolution of Δ*T*/*T*_0_ in Fig. 3D is attributed to the nonlinear thermodynamic modulation of the graphene properties illustrated in Fig. 1 and its related discussion above. It is also consistent with observations reported in numerous studies where the electron temperature dynamics were ascribed to heating-cooling effects using various techniques, including time-resolved angle-resolved photoemission spectroscopy (*22*), TPTP (*9*), optical pump–terahertz probe spectroscopy (*23*–*25*), and time-resolved photocurrent microscopy (*26*, *27*). We note here that the time resolution of our measurement, which is of the order of 2 ps, is limited by the noncollinear pump-probe geometry used in the experiment, as shown in Fig. 2A. The dependence of the peak values of Δ*T*/*T*_0_ on both the terahertz pump electric field and the gating voltage is shown in Fig. 3 (E and F). The as-measured data of Fig. 3E were obtained, for each gating voltage, via systematic variation of the terahertz peak field of the pump pulses using a pair of wire-grid polarizers. We see that Δ*T*/*T*_0_, i.e., the nonlinear response of graphene, increases with both the terahertz pump field and the gating voltage. We further see the saturation of Δ*T*/*T*_0_ setting in for gating voltages exceeding 0.7 V, which mimics the behavior of the graphene sheet resistance shown in Fig. 2D. We also note here that the data shown in Fig. 3 were collected for the negative branch of the gating voltage. On the positive branch of the gating voltage, the sample maintains a very similar (but not perfectly the same) behavior.

### Terahertz high harmonics

**Fig. 4:**
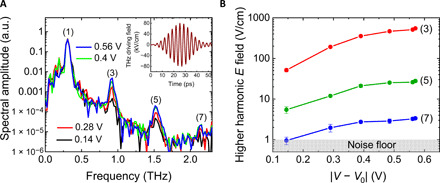
Gating dependence of terahertz HHG. (**A**) The terahertz amplitude spectra of the terahertz fields of the incident driving field and the transmitted fields through the graphene sample exhibiting generation of higher odd-order harmonics up to the seventh order for two doping levels, with the driving terahertz signal shown in the inset, and (**B**) the peak electric field of the generated harmonics as a function of the gating voltage.

In Fig. 4, we show the results of the nonlinear multicycle terahertz-TDS experiment using the same gated graphene sample. In this experiment, the sample was excited by a multicycle terahertz field with a central frequency of 0.3 THz, as shown in the inset of Fig. 4A. The peak electric field of the driving pulse was as high as 80 kV/cm, i.e., the same as the pump peak field in the single-cycle TPTP experiment. The response of the graphene sample was revealed through transmission measurements using calibrated free-space electro-optic sampling detection of the transmitted terahertz field. In Fig. 4A, we show the frequency-domain spectra of the terahertz pulses transmitted through the graphene sample at various gating voltages. The spectrum includes odd-order higher harmonics of the fundamental frequency *f*_1_ = 0.3 THz up to the seventh order, namely centered at the higher-harmonic frequencies *f*_3_ = 0.9 THz, *f*_5_ = 1.5 THz, and *f*_7_ = 2.1 THz. We note here that a conservative estimate of the corresponding nonlinear electronic susceptibilities for HHG from graphene in the perturbative regime (namely at lower driving terahertz fields below 30 kV/cm) are as high as χ^(3)^~10^−9^ m^2^/V^2^, χ^(5)^~10^−22^ m^4^/V^4^, and χ^(7)^~10^−38^ m^6^/V^6^, for the third-, fifth-, and seventh-order harmonics, respectively (*14*). These values are several orders of magnitude larger than the analogous coefficients of other known materials investigated in many different frequency ranges (*12*, *14*). As we see from Fig. 4A, the amplitudes of the generated harmonics, normalized to the peak at the fundamental frequency, are strongly enhanced by increasing the gating voltage from 0.14 to 0.56 V.

Figure 4B shows the dependence of the actual peak electric fields of the generated harmonics on the gating voltage, for a terahertz driving field of 80 kV/cm at the peak. Here, increasing the gating voltage from 0.14 to 0.56 V leads to a magnification of the generated harmonic fields by factors of 9 for the third harmonic, 4 for the fifth harmonic, and 3 for the seventh harmonic, which correspond to enhancement factors of 81, 16, and 9 for the power conversion efficiencies of these harmonics, respectively. These results display a wide range of tunability of the graphene nonlinearity and can pave ways for practical graphene-based ultrahigh-frequency electronics.

### Modeling the experimental results

**Fig. 5:**
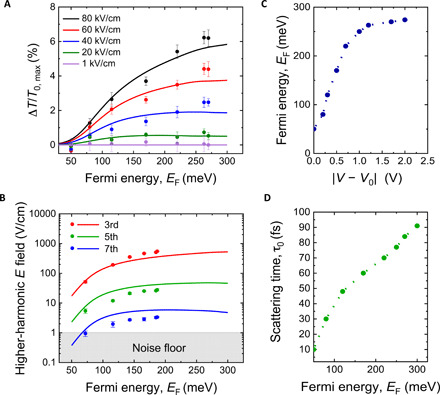
The thermodynamic model calculations. (**A** and **B**) The thermodynamic model calculations (solid lines) and the experimental results (solid circles) of the peak of Δ*T*/*T*_0_ and the electric field of the generated harmonics up to the seventh order, respectively, as functions of the *E*_F_ and various terahertz pump fields, (**C**) *E*_F_ as a function of the gating voltage, and (**D**) the carrier momentum scattering time as a function of *E*_F_.

We now reproduce the experimental results through simulations based on the thermodynamic model discussed above [see the Supplementary Materials and (*12*, *14*) for further information]. In our calculations, we simulate the nonlinear propagation of the driving terahertz signals, in the time domain, through the graphene layer at various Fermi energies corresponding to the experimentally tested gate voltage. In Fig. 5 (A and B), we show the model calculations (solid lines) along with the experimental results (symbols) of Figs. 3F and 4B, respectively, after replacing the gating voltage ∣*V* − *V*_0_∣ by the corresponding *E*_F_ shown in Fig. 5C. We see a good agreement between the experiment and the model calculations for the assumed range of *E*_F_ up to 200 meV, in which the graphene nonlinearity (both differential transmission and HHG) is enhanced substantially with increasing *E*_F_. Above this doping level and up to *E*_F_~260 meV, we see from Fig. 5A that the experimental data show saturation in the nonlinearity, which is also captured by the model calculations. We attribute this saturation behavior to the fact that the electronic heat capacity of graphene increases with *E*_F_ at high doping levels (*28*–*30*), restricting the increase in the carrier temperature with further increase in terahertz field excitation, which leads to a smaller increase in the graphene nonlinearity at these high Fermi energies (see the Supplementary Materials for further information). These effects are automatically included in the thermodynamic model by respecting the conservation of energy and total carrier density (*11*, *12*, *30*).

In our model calculations, we considered a range of *E*_F_ from 50 to 260 meV (as a variable parameter) to be equivalent to the values corresponding to the experimentally applied gating voltage ∣*V* − *V*_0_∣ in the range from 0 to 2 V, as shown in Fig. 5C. As previously noted, *E*_F_ is never uniformly zero at ∣*V* − *V*_0_∣ = 0. Therefore, we assigned a value of *E*_F_= 50 meV to the gating voltage ∣*V* − *V*_0_∣ = 0, which is consistent with the puddle energy estimates reported in previous works on similar graphene samples (*25*, *31*, *32*). The initial linear increase in *E*_F_ with the gating voltage is consistent with Hall measurements (see the Supplementary Materials). Another free parameter used in our calculations is the momentum scattering time τ_0_ associated with the linear response of graphene to low terahertz fields and is considered to be a function of *E*_F_ (*24*, *25*, *33*), as shown in Fig. 5D. Here, we considered the linear terahertz peak transmission *T*_0_ indicated in Fig. 3C, which is related to the “frequency-integrated conductivity” of the graphene layer, to estimate τ_0_ and *E*_F_ as carefully chosen pairs of values that reproduce the nonlinear response to the intense driving signals as well. We note that choosing other pairs of τ_0_ and *E*_F_ in our calculations resulted in a significant deviation between the model and the experimental data. With the obtained range of *E*_F_ and τ_0_, we extract a carrier mobility of ~3000 cm^2^/Vs, which is consistent with values reported for typical CVD graphene (*34*, *35*). Moreover, the increase in τ_0_ with *E*_F_ indicates that long-range Coulomb scattering dominates the carrier scattering process (*24*, *25*, *33*).

### Conclusions

The gigantic terahertz nonlinearity of graphene can be efficiently tuned by modest gating voltages. Our findings thus pave the way for exploiting graphene in applications that require a straightforward control of its nonlinear behavior. Therefore, applications such as graphene-CMOS hybrid devices for tunable frequency conversion, efficient nonlinear terahertz modulators, shutters, switches, and conceivably other applications, are all expected to benefit from both the gigantic nonlinearity of graphene and the possibility to efficiently tune it by a small gating voltage. Graphene also allows for electrical modulation at exceptionally high speeds, limited ultimately by the carrier cooling time of a few picoseconds, thus corresponding to a bandwidth of a few hundreds of gigahertz. Graphene-based amplitude (*36*) and phase (*37*) modulators with bandwidths as large as a few tens of gigahertz have been already demonstrated. Whereas these speeds cannot be reached with the polymer electrolyte that we used here, it is attainable for example with a doped silicon back gate or by hybridizing the electrolyte-gated graphene with back-gating (*38*). This opens prospects for efficient nonlinear conversion of terahertz signals that can be modulated with a bandwidth of tens of gigahertz or even higher, which is of great interest for ultrahigh-speed information and communication technologies.

## MATERIALS AND METHODS

In the TPTP experiment, intense single-cycle terahertz pulses with a peak electric field strength up to 80 kV/cm are generated by optical rectification of 800 nm, 40 fs Ti:sapphire laser pulses in a LiNbO_3_ crystal, following the pulse-front tilting technique (*39*, *40*), and are used as the pump pulses to excite the graphene sample. The terahertz probe pulses, with a much weaker peak electric field of less than 1 kV/cm, are generated by optical rectification of 800-nm pulses from the same Ti:sapphire laser in a 1-mm-thick (110) ZnTe crystal. Both pump and probe pulses are detected by free-space electro-optic sampling in another 1-mm-thick (110) ZnTe crystal. Spatial separation between the pump and probe beams after the graphene sample was attained by following a noncolinear incidence onto the sample. Combined with a cross-polarization scheme (s-polarization for the intense pump beam and p-polarization for the weak probe), this sufficiently suppressed the intense pump beam after the sample to allow for a sensitive detection of the weak probe pulses that carry the desired information about the pump-induced effects in graphene.

In the multicycle experiments, the graphene sample was excited by terahertz pulses generated by the accelerator-based TELBE superradiant source (*41*, *42*), with a central fundamental frequency of 0.3 THz, as shown in Figs. 2B and 4A. The incident and transmitted terahertz pulses through the graphene sample have been detected by electro-optic sampling in a 1.9-mm-thick (110) ZnTe crystal. To enable sensitive detection of the generated harmonics and reduce the dominating driving field at the detection point, a set of high-pass filters (not shown here) was used after the graphene sample. This experiment, except for the gated graphene sample examined here, is analogous to that reported in (*14*), and further details about this experiment are found therein.

The gate-tunable graphene sample, as shown in Fig. 2C, was prepared as follows: (i) We used commercial monolayer graphene (from Graphenea) prepared via CVD on copper foil. (ii) Using wet transfer, we transferred a large (~1 cm by 1 cm) piece of graphene on an Infrasil quartz substrate. (iii) We then used optical lithography to define the source and drain contacts to the graphene, as well as two metal electrodes on the side, which are close to graphene without touching it. For all electrodes, we used thermal evaporation of titanium-gold. (iv) Then, we drop-casted a transparent polymer electrolyte top gate, consisting of PEO and LiClO_4_ with 8:1 weight ratio in a solution of methanol (*43*) on top of the graphene and the two gate electrodes. Last, we glued the silica substrate onto a home-built printed circuit board (PCB) with a large hole in the center to allow for optical transmission measurements and wire-bonded the six electrodes to six leads connected to SMA connectors. This allowed us to measure the graphene channel resistance using the electrodes in contact with graphene, while being able to electrically tune the Fermi energy by changing the voltage on the two gate electrodes (see also the Supplementary Materials for Hall measurements of a similar device).

## SUPPLEMENTARY MATERIALS

Supplementary material for this article is available at http://advances.sciencemag.org/cgi/content/full/7/15/eabf9809/DC1

## References

[1] V. G. Bozhkov, Semiconductor detectors, mixers, and frequency multipliers for the terahertz band. Radiophys. Quantum Electron. 46, 631–656 (2003).

[2] A. Maestrini, J. S. Ward, J. J. Gill, C. Lee, B. Thomas, R. H. Lin, G. Chattopadhyay, I. Mehdi, A frequency-multiplied source with more than 1 mW of power across the 840–900-GHz band. IEEE Trans. Microw. Theory Tech. 58, 1925–1932 (2010).

[3] J. V. Siles, J. Grajal, Physics-based design and optimization of schottky diode frequency multipliers for terahertz applications. IEEE Trans. Microw. Theory Tech. 58, 1933–1942 (2010).

[4] F. Schwierz, Graphene transistors: Status, prospects, and problems. Proc. IEEE 101, 1567–1584 (2013).

[5] H. Wang, D. Nezich, J. Kong, T. Palacios, Graphene frequency multipliers. IEEE Electron Device Lett. 30, 547–549 (2009).

[6] D. Neumaier, S. Pindl, M. C. Lemme, Integrating graphene into semiconductor fabrication lines. Nat. Mater. 18, 525–529 (2019). 31114067 10.1038/s41563-019-0359-7

[7] K. S. Novoselov, A. K. Geim, S. V. Morozov, D. Jiang, M. I. Katsnelson, I. V. Grigorieva, S. V. Dubonos, A. A. Firsov, Two-dimensional gas of massless Dirac fermions in graphene. Nature 438, 197–200 (2005). 16281030 10.1038/nature04233

[8] A. K. Geim, K. S. Novoselov, The rise of graphene. Nat. Mater. 6, 183–191 (2007). 17330084 10.1038/nmat1849

[9] H. Y. Hwang, N. C. Brandt, H. Farhat, A. L. Hsu, J. Kong, K. A. Nelson, Nonlinear THz conductivity dynamics in p-type CVD-grown graphene. J. Phys. Chem. B 117, 15819–15824 (2013). 24102144 10.1021/jp407548a

[10] M. J. Paul, Y. C. Chang, Z. J. Thompson, A. Stickel, J. Wardini, H. Choi, E. D. Minot, B. Hou, J. A. Nees, T. B. Norris, Y. S. Lee, High-field terahertz response of graphene. New J. Phys. 15, 085019 (2013).

[11] Z. Mics, K. J. Tielrooij, K. Parvez, S. A. Jensen, I. Ivanov, X. Feng, K. Müllen, M. Bonn, D. Turchinovich, Thermodynamic picture of ultrafast charge transport in graphene. Nat. Commun. 6, 7655 (2015). 26179498 10.1038/ncomms8655 PMC4518297

[12] H. A. Hafez, S. Kovalev, K.-J. Tielrooij, M. Bonn, M. Gensch, D. Turchinovich, Terahertz nonlinear optics of graphene: From saturable absorption to high-harmonics generation. Adv. Opt. Mater. 8, 1900771 (2019).

[13] P. Bowlan, E. Martinez-Moreno, K. Reimann, T. Elsaesser, M. Woerner, Ultrafast terahertz response of multilayer graphene in the nonperturbative regime. Phys. Rev. B 89, 041408 (2014).

[14] H. A. Hafez, S. Kovalev, J. C. Deinert, Z. Mics, B. Green, N. Awari, M. Chen, S. Germanskiy, U. Lehnert, J. Teichert, Z. Wang, K. J. Tielrooij, Z. Liu, Z. Chen, A. Narita, K. Müllen, M. Bonn, M. Gensch, D. Turchinovich, Extremely efficient terahertz high-harmonic generation in graphene by hot Dirac fermions. Nature 561, 507–511 (2018). 30202091 10.1038/s41586-018-0508-1

[15] J.-C. Deinert, D. A. Iranzo, R. Pérez, X. Jia, H. A. Hafez, I. Ilyakov, N. Awari, M. Chen, M. Bawatna, A. N. Ponomaryov, S. Germanskiy, M. Bonn, F. H. L. Koppens, D. Turchinovich, M. Gensch, S. Kovalev, K.-J. Tielrooij, Grating-graphene metamaterial as a platform for terahertz nonlinear photonics. ACS Nano 15, 1145–1150 (2021). 33306364 10.1021/acsnano.0c08106 PMC7844822

[16] F. Schwierz, Industry-compatible graphene transistors. Nature 472, 41–42 (2011). 21475186 10.1038/472041a

[17] S. Goossens, G. Navickaite, C. Monasterio, S. Gupta, J. J. Piqueras, R. Pérez, G. Burwell, I. Nikitskiy, T. Lasanta, T. Galán, E. Puma, A. Centeno, A. Pesquera, A. Zurutuza, G. Konstantatos, F. Koppens, Broadband image sensor array based on graphene–CMOS integration. Nat. Photonics 11, 366–371 (2017).

[18] D. G. Paveliev, Y. I. Koshurinov, A. S. Ivanov, A. N. Panin, V. L. Vax, V. I. Gavrilenko, A. V. Antonov, V. M. Ustinov, A. E. Zhukov, Experimental study of frequency multipliers based on a GaAs/AlAs semiconductor superlattices in the terahertz frequency range. Sem. Ther. 46, 121–125 (2012).

[19] I. Mehdi, J. V. Siles, C. Lee, E. Schlecht, THz diode technology: Status, prospects, and applications. Proc. IEEE 105, 990–1007 (2017).

[20] A. Pirkle, J. Chan, A. Venugopal, D. Hinojos, C. W. Magnuson, S. McDonnell, L. Colombo, E. M. Vogel, R. S. Ruoff, R. M. Wallace, The effect of chemical residues on the physical and electrical properties of chemical vapor deposited graphene transferred to SiO_2_. Appl. Phys. Lett. 99, 122108 (2011).

[21] S. Ryu, L. Liu, S. Berciaud, Y. J. Yu, H. Liu, P. Kim, G. W. Flynn, L. E. Brus, Atmospheric oxygen binding and hole doping in deformed graphene on a SiO_2_ substrate. Nano Lett. 10, 4944–4951 (2010). 21069971 10.1021/nl1029607

[22] I. Gierz, J. C. Petersen, M. Mitrano, C. Cacho, I. C. E. Turcu, E. Springate, A. Stöhr, A. Köhler, U. Starke, A. Cavalleri, Snapshots of non-equilibrium Dirac carrier distributions in graphene. Nat. Mater. 12, 1119–1124 (2013). 24097235 10.1038/nmat3757

[23] G. Jnawali, Y. Rao, H. Yan, T. F. Heinz, Observation of a transient decrease in terahertz conductivity of single-layer graphene induced by ultrafast optical excitation. Nano Lett. 13, 524–530 (2013). 23330567 10.1021/nl303988q

[24] K. J. Tielrooij, J. C. W. Song, S. A. Jensen, A. Centeno, A. Pesquera, A. Zurutuza Elorza, M. Bonn, L. S. Levitov, F. H. L. Koppens, Photoexcitation cascade and multiple hot-carrier generation in graphene. Nat. Phys. 9, 248–252 (2013).

[25] A. J. Frenzel, C. H. Lui, Y. C. Shin, J. Kong, N. Gedik, Semiconducting-to-metallic photoconductivity crossover and temperature-dependent drude weight in graphene. Phys. Rev. Lett. 113, 056602 (2014). 25126929 10.1103/PhysRevLett.113.056602

[26] K. J. Tielrooij, L. Piatkowski, M. Massicotte, A. Woessner, Q. Ma, Y. Lee, K. S. Myhro, C. N. Lau, P. Jarillo-Herrero, N. F. van Hulst, F. H. L. Koppens, Generation of photovoltage in graphene on a femtosecond timescale through efficient carrier heating. Nat. Nanotechnol. 10, 437–443 (2015). 25867941 10.1038/nnano.2015.54

[27] M. W. Graham, S. F. Shi, D. C. Ralph, J. Park, P. L. McEuen, Photocurrent measurements of supercollision cooling in graphene. Nat. Phys. 9, 103–108 (2013).

[28] J. C. W. Song, M. Y. Reizer, L. S. Levitov, Disorder-assisted electron-phonon scattering and cooling pathways in graphene. Phys. Rev. Lett. 109, 106602 (2012). 23005313 10.1103/PhysRevLett.109.106602

[29] S.-F. Shi, T. T. Tang, B. Zeng, L. Ju, Q. Zhou, A. Zettl, F. Wang, Controlling graphene ultrafast hot carrier response from metal-like to semiconductor-like by electrostatic gating. Nano Lett. 14, 1578–1582 (2014). 24564302 10.1021/nl404826r

[30] S. A. Jensen, Z. Mics, I. Ivanov, H. S. Varol, D. Turchinovich, F. H. L. Koppens, M. Bonn, K. J. Tielrooij, Competing ultrafast energy relaxation pathways in photoexcited graphene. Nano Lett. 14, 5839–5845 (2014). 25247639 10.1021/nl502740g

[31] H. A. Hafez, P. L. Lévesque, I. al-Naib, M. M. Dignam, X. Chai, S. Choubak, P. Desjardins, R. Martel, T. Ozaki, Intense terahertz field effects on photoexcited carrier dynamics in gated graphene. Appl. Phys. Lett. 107, 251903 (2015).

[32] H. Razavipour, W. Yang, A. Guermoune, M. Hilke, D. G. Cooke, I. al-Naib, M. M. Dignam, F. Blanchard, H. A. Hafez, X. Chai, D. Ferachou, T. Ozaki, P. L. Lévesque, R. Martel, High-field response of gated graphene at terahertz frequencies. Phys. Rev. B 92, 245421 (2015).

[33] S. Das Sarma, S. Adam, E. H. Hwang, E. Rossi, Electronic transport in two-dimensional graphene. Rev. Mod. Phys. 83, 407–470 (2011).

[34] X. Li, W. Cai, J. An, S. Kim, J. Nah, D. Yang, R. Piner, A. Velamakanni, I. Jung, E. Tutuc, S. K. Banerjee, L. Colombo, R. S. Ruoff, Large-area synthesis of high-quality and uniform graphene films on copper foils. Science 324, 1312–1314 (2009). 19423775 10.1126/science.1171245

[35] D. S. L. Abergel, V. Apalkov, J. Berashevich, K. Zieglerc, T. Chakraborty, Properties of graphene: A theoretical perspective. Adv. Phys. 59, 261–482 (2010).

[36] C. T. Phare, Y. H. Daniel Lee, J. Cardenas, M. Lipson, Graphene electro-optic modulator with 30 GHz bandwidth. Nat. Photonics 9, 511–514 (2015).

[37] V. Sorianello, M. Midrio, G. Contestabile, I. Asselberghs, J. van Campenhout, C. Huyghebaert, I. Goykhman, A. K. Ott, A. C. Ferrari, M. Romagnoli, Graphene-silicon phase modulators with gigahertz bandwidth. Nat. Photonics 12, 40–44 (2018).

[38] D. Cano, A. Ferrier, K. Soundarapandian, A. Reserbat-Plantey, M. Scarafagio, A. Tallaire, A. Seyeux, P. Marcus, H. de Riedmatten, P. Goldner, F. H. L. Koppens, K. J. Tielrooij, Fast electrical modulation of strong near-field interactions between erbium emitters and graphene. Nat. Commun. 11, 4094 (2020). 32796825 10.1038/s41467-020-17899-7 PMC7427803

[39] J. Hebling, G. Almási, I. Z. Kozma, J. Kuhl, Velocity matching by pulse front tilting for large area THz-pulse generation. Opt. Express 10, 1161–1166 (2002). 19451975 10.1364/oe.10.001161

[40] H. Hirori, A. Doi, F. Blanchard, K. Tanaka, Single-cycle terahertz pulses with amplitudes exceeding 1 MV/cm generated by optical rectification in LiNbO3. Appl. Phys. Lett. 98, 091106 (2011).

[41] B. Green, S. Kovalev, V. Asgekar, G. Geloni, U. Lehnert, T. Golz, M. Kuntzsch, C. Bauer, J. Hauser, J. Voigtlaender, B. Wustmann, I. Koesterke, M. Schwarz, M. Freitag, A. Arnold, J. Teichert, M. Justus, W. Seidel, C. Ilgner, N. Awari, D. Nicoletti, S. Kaiser, Y. Laplace, S. Rajasekaran, L. Zhang, S. Winnerl, H. Schneider, G. Schay, I. Lorincz, A. A. Rauscher, I. Radu, S. Mährlein, T. H. Kim, J. S. Lee, T. Kampfrath, S. Wall, J. Heberle, A. Malnasi-Csizmadia, A. Steiger, A. S. Müller, M. Helm, U. Schramm, T. Cowan, P. Michel, A. Cavalleri, A. S. Fisher, N. Stojanovic, M. Gensch, High-field high-repetition-rate sources for the coherent THz control of matter. Sci. Rep. 6, 22256 (2016). 26924651 10.1038/srep22256 PMC4770290

[42] S. Kovalev, B. Green, T. Golz, S. Maehrlein, N. Stojanovic, A. S. Fisher, T. Kampfrath, M. Gensch, Probing ultra-fast processes with high dynamic range at 4th-generation light sources: Arrival time and intensity binning at unprecedented repetition rates. Struct. Dyn. 4, 024301 (2017). 28382317 10.1063/1.4978042 PMC5346102

[43] A. Das, S. Pisana, B. Chakraborty, S. Piscanec, S. K. Saha, U. V. Waghmare, K. S. Novoselov, H. R. Krishnamurthy, A. K. Geim, A. C. Ferrari, A. K. Sood, Monitoring dopants by Raman scattering in an electrochemically top-gated graphene transistor. Nat. Nanotechnol. 3, 210–215 (2008). 18654505 10.1038/nnano.2008.67

[44] I. Gierz, F. Calegari, S. Aeschlimann, M. Chávez Cervantes, C. Cacho, R. T. Chapman, E. Springate, S. Link, U. Starke, C. R. Ast, A. Cavalleri, Tracking primary thermalization events in graphene with photoemission at extreme time scales. Phys. Rev. Lett. 115, 086803 (2015). 26340199 10.1103/PhysRevLett.115.086803

